# Modifiable dementia risk factors in Chilean adults are distinctively associated with social determinants of health. Cross-sectional study

**DOI:** 10.1186/s12889-025-22220-6

**Published:** 2025-03-24

**Authors:** Juan José Mariman, Rodrigo C. Vergara, Consuelo San Martin, Victor Zapata, Oscar Arteaga, Paul H. Delano, Carolina Delgado Derio

**Affiliations:** 1 Centro de Investigación (CEI-UMCE), Universidad Metropolitana de Ciencias de la Educación, Santiago, Chile; 2 Departamento de Kinesiología, Facultad de Artes y Educación Física, Universidad Metropolitana de Ciencias de la Educación, Santiago, Chile; 3 Departamento de Kinesiología, Facultad de Medicina, Universidad de Chile, Santiago, Chile; 4 Centro Nacional de Inteligencia Artificial, Santiago, Chile; 5 Universidad de los Andes, Escuela de Psicología, Santiago, Chile; 6 Gerencia de Operaciones. Hospital Clínico Universidad de Chile, Santiago, Chile; 7 Escuela de Salud Pública, Facultad de Medicina, Universidad de Chile, Santiago, Chile; 8 Servicio Otorrinolaringología, Hospital Clínico de la Universidad de Chile, Santiago, Chile; 9 Centro Avanzado de Ingeniería Eléctrica y Electrónica, Universidad Técnica Federico, Santiago, AC3E Chile; 10 Departamento de Neurociencia, Facultad de Medicina, Universidad de Chile, Santiago, Chile; 11 Departamento de Neurología y Neurocirugía. Hospital Clínico, Unidad de Cerebro Saludable, Universidad de Chile, Santiago, Chile

**Keywords:** Dementia, Prevention, Risk factors, Clusters, Latin America, Metabolic, Cardiovascular, Behavioral, Depression, Socioeconomic, Multimorbidity

## Abstract

**Background:**

In Latin America, dementia cases will double by 2050. For effective prevention in this region, it is crucial to comprehend the distribution of dementia risk factors within the local population and to assess their association with social determinants of health (SDH). Our objective was to explore the association between different modifiable dementia risk factors within the Chilean population in a cross-sectional study.

**Methods:**

3379 dementia-free subjects ≥ 45 years old from the 2016–2017 Chilean National Health Survey were analyzed and stratified into four groups by sex and age, searching for clusters using six continuous variables that had been related to dementia risk (years of education, systolic blood pressure, body mass index (BMI), units of alcohol consumption, physical activity, and depressive symptoms).

**Results:**

Three clusters of individuals shared similar risk factors in each sex/age group. A cluster with high cardiometabolic risk was present in all sex/age groups, characterized by high systolic blood pressure (HSBP) in men midlife and by HSBP associated with high BMI (HSBP/HBMI) in women and in men later-life. A depressive cluster and a physically inactive cluster were present in 3⁄4 of the sex/age groups. Additionally, there was a cluster that was relatively healthy but had a risk of excessive alcohol consumption in men later-life and a low risk one in women midlife. The HSBP/HBMI and depressive clusters presented a high proportion of multiple dementia risk factors. Lower levels of education (and lower family income) were associated with the HSBP and HSBP/HBMI cluster; in contrast, higher levels of education were associated with clusters with lower risk.

**Conclusion:**

In Chile, subpopulations with more disadvantages SDH have a high prevalence of cardiometabolic risk factors. Subpopulations with depression and those with high cardiometabolic risk have a higher accumulation of dementia risk factors. These results highlight that tailored programs improving healthcare accessibility for those with more disadvantages SDH and multidisciplinary interventions for high-risk populations are needed for effective dementia prevention.

**Supplementary Information:**

The online version contains supplementary material available at 10.1186/s12889-025-22220-6.

## Introduction

Latin America and the Caribbean (LAC) regions encompass multiple countries with diverse cultural backgrounds, multiracial and multiethnic populations, and varying levels of the human development index [1]. Most of these countries are experiencing an advanced epidemiological transition due to the accelerated aging of the last few decades [1]. Closely related to aging, the number of people living with dementia is forecasted to increase between 80% and 240% in the next three decades in LAC countries, which is higher than the projections for the US or European countries [2]. However, these projections underestimate the effects of possible changes in the exposure to modifiable risk factors for dementia over time, which account for 45% ^3^ to 56% ^4–6^of the dementia cases attributed to these risk factors in LAC, a higher proportion than those estimated in other world regions [3–8] Therefore, there is a great opportunity for improving dementia prevention in LACs. Unfortunately, research on dementia-modifiable risk factors in this region is very scarce [9]. The available data from cross-sectional studies show that some LAC countries have a higher prevalence of cardiovascular risk factors and lower educational levels than other regions [3, 4, 6].

Additionally, one longitudinal multicountry-based cohort from 2003 showed that higher cardiovascular risk factors were associated with increased dementia risk, especially in those with lower income [10]. There are multiple individual modifiable risk factors associated with higher dementia risk [11], and many of them coexist in the same population, with multimorbidity being very common in adults and increasing with aging; thus, integral preventive strategies should focus on multiple health conditions [12, 13]. In this context, previous research has shown a dose effect between the number of dementia risk factors and the development of dementia, but the interactions among dementia risk factors have been poorly studied [12].

It has been shown that the presence of multiple risk factors in the population tends to form clusters or subgroups of individuals who share similar risk factors, being the most common clusters related to cardiometabolic diseases (i.e., a subgroup of persons with two or more cardiovascular risk factors or diseases), mental health problems and musculoskeletal conditions [14]. Additionally, the coexistence of multiple risk factors is more common in individuals belonging to lower social classes, such as those with lower levels of education and wealth [14]. These last nonmedical variables related to where people are born, grow, work, live, and age are called social determinants of health (SDH) [15]. In fact, recent Brazilian population-based studies have shown that the poorest regions of Brazil have a higher proportion of dementia risk factors than the richest regions [4], and Black and Brown ethnicities are more susceptible to SDH than white people [5]. In Argentina, the poorest quintile of the population had a greater proportion of the population attributable fraction of dementia risk factors than the richest one [8].

Thus, it seems crucial for dementia prevention in LAC, considering its cultural diversity, to explore locally the presence and interactions of dementia risk factors, including the SDH [1], because it will allow the development of tailored interventions for certain subgroups of society [15, 16]. Chile is a valuable LAC country for examining the relationship between SDH and the prevalence of dementia risk factors because noncommunicable diseases are the main cause of DALYs [17] and have significant socioeconomic disparities. Our hypothesis is that in Chile, specific dementia risk factors are associated between them and grouped into clusters of persons who share similar SDHs.

In this study, we aim to investigate in a nationally representative sample of non-demented Chilean adults aged 45 years and older whether dementia risk factors can be grouped into population profiles and analyze their association with SDH. To do so, we focused on the presence of adult cardiometabolic, behavioral, and mental health conditions and used the educational level as a pivotal SDH in our clustering analysis.

## Methods

### Study design

This is a cross-sectional study where we analyzed the data from the last Chilean National Health Survey (Ch-NHS) carried out between October 2016 and March 2017, which collected health information from a representative sample of Chileans over 15 years of age. The sample design was probabilistic, geographically stratified, and multistage. It included 6,233 participants (household response rate 66%, rejection rate 9.8%) who were interviewed during a first visit using structured questionnaires. In a second visit, anthropometric and laboratory measurements were performed by trained personnel. All subjects gave written informed consent in accordance with the Declaration of Helsinki, monitored by the Scientific Ethics Committee of the Universidad Católica de Chile [18]. To account for an adequate representation of population groups, weighting factors are calculated based on a 4-stage probabilistic analysis that allows the selection of a particular subject for a house that belongs to a block or locality within a stratum of communes (the Chilean territory was divided into 30 strata of communes). In each step, an adjusted conditional probability approximation was used. This allows an individual expansion factor to be determined for each sample. More details can be seen on the Chilean Health Ministry Website [19].

### Subject selection

Our analysis included subjects ≥ 45 years old, as this group presented the highest risk of developing dementia within the next 20–30 years. To further increase the precision of clustering across different subpopulations, we stratified our analysis by sex and age (< and > to 65 years old). We excluded from the total sample those participants with suspected cognitive impairment by using the shortened Chilean version of the mini-mental state examination [3, 17, 18].

### Dementia risk factors and population risk subgroups

The Ch-NHS has data on 9 of the 12 established dementia risk factors (low education, hearing loss, hypertension, alcohol consumption, obesity, smoking, diabetes, depression, and physical inactivity) [3, 11]. Because the main aim of the cluster analysis was to detect groups of people with similar risk factors in the most precise and sensitive way and to capture their full range of variation, we chose only variables measured with continuous scales and, whenever possible, with objective measurements instead of dichotomic self-report variables [17] (which is why we excluded hearing loss, diabetes, and smoking from cluster analysis). We selected six variables representative of socioeconomic, behavioral, metabolic, and mental health conditions [14, 17] that were the educational level, systolic blood pressure (SBP), body mass index (BMI), physical activity, units of alcohol consumption, and depressive symptoms (described in e-Table 1 in supplement [20]).

Finally, to be representative of the Chilean population, we included only those with > 85% completed cases in the Ch-NHS.

#### Cluster characterization

We characterize each cluster using different approaches. First, we used the descriptive data of the six variables for clustering. Furthermore, for a general overview of the total dementia risk ^3, 11,^ we also described the data of the nine dementia risk factors, comparing their co-occurrence between clusters (the six variables used for clustering, plus hearing loss, smoking, and diabetes). Finally, for a sensitivity analysis of the main characteristics of each cluster, we also included (1) the fasting plasma glucose level as an alternative measurement of glucose metabolism (in addition to diabetes), (2) the waist circumference as an alternative nutritional assessment (in addition to BMI) and (3) the total monthly family income (in USD$) as an alternative measurement of SDH (in addition to educational level). Moreover, we describe the proportion of “metabolic syndrome”, a variable that considers the accumulation of several metabolic risk factors. In Table 1, we describe the 11 categories of variables used in cluster characterization, including their definitions as continuous and dichotomic variables when appropriate (education, income, blood pressure, nutritional status, glucose metabolism, metabolic syndrome, depressive symptoms, hearing loss, OH consumption, smoking, and physical activity).

### Statistical analysis

All analyses were performed using the statistical software R project, considering the weights of the sample design and the maximum complete cases available [20]. No imputation procedure was used for missing data. All the analyses were stratified by sex (women/men) and two age ranges: 45–65 years old or “midlife” and older than 65 years or “later-life” [11]. This approach may diminish the underrepresentation of those in later life in cluster analysis.

#### Cluster analysis

##### Detection of subgroups

With the motivation to detect population profiles according to the score of the six continuous variables, we performed cluster analysis. Cluster analysis groups observations (i.e., people) according to their differences and similarities in selected characteristics (six continuous variables described in Sect. 2.3.1). As such, the algorithm produced the most different groups of people with the most similar people within each group, given the variables used to explore such differences. Among different clustering algorithms, we selected the “Partitioning around medoids” (PAM) clustering [21]. PAM clustering seeks to find groups of observations around a data point (named “medoids”) to minimize the dissimilarity between them and respect the medoid. In comparison with the traditional K-means algorithm, which uses the Euclidean distance to calculate the (dis)similarity between the observations, the PAM method offers different options for the distance matrix calculation (Euclidean, Manhattan, Pearson, etc.), giving a better chance to fit the algorithm to the nature of the data. Additionally, the PAM chooses the medoid (the center of the cluster) from the observation itself, which improves the interpretation of the results. Finally, in the R project, the PAM method considers the weight of each sample for cluster computation (which, in our case, allows the usage of survey weights of the Ch-NHS and reduces the computational time for the weighted Ch-NHS variable). The complete pipeline included data standardization using the z score, exploration of clustering solutions in a range of 3 to 10 clusters based on the Euclidean distance and ward D2 method, computation of PAM clustering and selection of the solution that accomplished the following criteria: (1) higher silhouette and Dunn indices, (2) cluster conformation such that the difference between the size of the clusters (ratio) was lower than 0.5 to obtain meaningful grouping, and (3) the lowest misidentified data according to the silhouette method. Once profiles were obtained at each sex/age group, we characterized them based on the six variables used to produce the clusters. We named each cluster using the name of the metabolic, behavioral, and/or mental health variables instead of the educational level because the first ones represent the adult’s present health state, in contrast to the educational level that represents childhood and was used for performing the socioeconomic characterization of each cluster. For naming, we considered the variable with (1) the least healthy value and (2) the variable with the largest difference between the clusters. As an example of the first condition, although most of the clusters had low physical activity, the “physically inactive” cluster presented low physical inactivity as their most unhealthy variable, while the other clusters had other unhealthier characteristics. As an example of the second condition, although the depressive cluster in late-life women also had very high metabolic risk, depressive symptoms were the most distinctive characteristic compared with the other clusters.

#### Cluster characterization and association with SDH

We characterized each cluster by describing each dementia risk factor as a continuous variable (mean ± 95% CI) and a dichotomous variable (the proportion %, ± 95% CI) within each cluster. For the contrast analysis, we compared if there were significant differences (no superposition of confidence intervals) in each of these values between the clusters. Specifically, for SDH, we compared the level of education and the total family income between clusters. Also, we compared the total number of dementia risk factors between them.

## Results

### Demographic

From 3379 participants aged ≥ 45 years old without cognitive impairment, we excluded 501 participants (15% of the sample) that have missing data in any of the six variables chosen for the cluster analysis, leaving us with 2878 people, with 1867 women and 1011 men who represented 65% and 35% of the sample, respectively.

### Excluded participants

 Midlife men were slightly underrepresented compared to the Chilean census proportions in 2017 (Table 1), likely due to many being employed and thus unable to undergo physical examinations (SBP and BMI) and laboratory tests (glucose levels) during the second visit of the Ch-NHS. To assess the impact of their exclusion, self-reported data from both included and excluded participants were compared (Table 2). Excluded midlife men exhibited higher education levels, smoking rates, and depression rates, with no significant differences in self-reported hypertension or diabetes compared to included men. Excluded midlife women have lower education levels and smoking rates but showed a tendency towards higher self-reported hypertension and diabetes. In later life, excluded participants had lower rates of self-reported hearing loss and smoking but higher rates of physical inactivity. They also tended to have lower proportions of self-reported diabetes and hypertension.”

**Table 1:** Demographic characteristics

Sex	Age	Ch-NHS included Participants	Ch-NHS Weighted Numbers	Ch-NHS sex/age proportions	Chilean total population* sex/age proportions
Women	Midlife (45–64)	1,135	2,001,107	11.38%	12%
	Later life (≥ 65)	732	837,765	4.76%	6%
Men	Midlife (45–64)	607	1,726,082	9.82%	12%
	Later life (≥ 65)	404	654,252	3.72%	5%

* Chilean proportion of women and men ≥ 45 years old according to the 2017 census

**Table 2:** Comparison of 9 dementia risk factors between clusters

Women Midlife	High Metabolic Risk	Depressive	Low Risk
Years of education	**7.3 (6.8–7.8)**	10.8 (10.1–11.5)	11.9 (11.5–12.3)
Low education (< 7 years) (%)	**43.0 (32.6–53.4)**	7.6 (1.1–14.1)	2.9 (1.1–4.7)
Systolic blood pressure (mmHg)	**134.2 (130.6–137.8)**	120.4 (116.6–124.2)	122.9 (120.8–125.0)
High systolic blood pressure (%)	32.2 (22.5–41.8)	14.4 (1.7–27.0)	13.4 (8.3–18.4)
Body Mass Index (BMI)	**33.3 (32.6–34.1)**	**30.5 (29.1–32.0)**	**27.6 (27.0–28.2)**
Obesity (BMI > 30) (%)	**73.2 (60.3–86.1)**	45.7 (29.2–62.3)	**24.1 (15.3–32.8)**
Diabetes Mellitus (%)	**27.6 (20–35.2)**	23.5 (11.9–35.2)	**10.7 (4.9–16.5)**
N° of Depressive symptoms	0.7 (0.3–1.1)	**5.9 (5.3–6.4)**	0.2 (0.1–0.3)
Major Depression (%)	10.8 (3.4–18.2)	**90.0 (67.7–112.4)**	3.6 (1.1–6.2)
Hearing loss (%)	20.2 (13.3–27.2)	34.7 (19.0–50.3)	19.6 (13.4–25.8)
Alcohol units per week	0.8 (0.5–1.1)	2.1(0.6–3.5)	1.2 (0.7–1.7)
OH excess (%)	0.0 (0–0)	**3.7 (1.5–8.9)**	0.2 (0.2–0.6)
Current smoker (%)	23.5(15.8–31.2)	42.3 (28.2–56.5)	30.9 (21.6–40.1)
Physical activity per week (Mets)	509.2 (365.9–652.4)	883.6 (606.5–1160.7)	**1580.9 (1329.8–1832.0)**
Physical inactivity (%)	**72.1 (60.5–83.7)**	56.7 (38.1–75.3)	**44.2 (35.2–53.2)**
**Women Later life**	**High Metabolic Risk**	**Depressive**	**Physically Inactive**
Years of education	**4.7 (4.2–5.2)**	**6.3 (5.5–7.1)**	**10.6 (9.7–11.6)**
Low education (< 7 years) (%)	**78.0 (65.0–91.1)**	**51.8 (38.1–65.4)**	**21.2 (13.2–29.1)**
Systolic blood pressure (mmHg)	**157.1 (153.2–160.9)**	**143.71(138.6 -148.7)**	**122.86(119.9–125.7)**
High systolic blood pressure (%)	**78.3 (65.8–90.8)**	**49.6 (33.4–65.7)**	**11.5 (4.0–18.9)**
Body Mass Index (BMI)	29.9 (28.9–31.0)	29.5(28.2–30.8)	28.6 (27.6–29.6)
Obesity (BMI > 30) (%)	49.3 (38.1–60.6)	40.9(22.5–59.4)	37.8(25.6–49.9)
Diabetes Mellitus (%)	33.9 (22.6–45.3)	44.3 (26.7–61.8)	27.4 (18.1–36.7)
N° of Depressive symptoms	0.1 (0.04–0.2)	**5.9 (5.5–6.2)**	0.4 (0.08–0.7)
Major Depression (%)	0.3 (-0.3–1.0)	**99.0(78.0–120.1)**	8.2 (0.8–15.7)
Hearing loss (%)	41.4 (29.4–53.4)	48.1(29.6–66.7)	43.5 (29.6–57.5)
Alcohol units per week	0.8 (0.5–1.1)	0.6(0.1–1.1)	1.6 (0.8–2.4)
OH excess (%)	0.06 (0.06–0.19)	0 (0–0)	1.0 (0.9–3.0)
Current smoker (%)	7.1 (3.2–10.9)	8.9 (1.7–16.1)	16.4 (6.8–26.0)
Physical activity per week (Mets)	670.3 (461.1–879.5)	705.2 (352.9–1057.6)	479.4 (334.9–624.0)
Physical inactivity (%)	72.6 (59.5–85.6)	66.9 (48.3–85.5)	72.8 (56.6–89.1)
**Men midlife**	**High Systolic Blood Pressure**	**Depressive**	**Physically Inactive**
Years of education	7.8 (7.2–8.4)	9.7 (7.8–11.7)	**12.4 (11.9–13.0)**
Low education (< 7 years) (%)	29.7 (20.0–39.4)	11.2 (5.9–28.4)	**2.1 (0.3–4.0)**
Systolic blood pressure (mmHg)	143.1 (137.9–148.4)	135.8 (132.5–139.1)	**125.6 (123.2–128.0)**
High systolic blood pressure (%)	48.2 (35.5–60.9)	24.1 (10.1–38.0)	**11.8 (7.3–16.3)**
Body Mass Index (BMI)	**27.6 (26.9–28.2)**	29.2 (27.0–31.5)	**29.8 (29.0–30.5)**
Obesity (BMI > 30) (%)	**20.9 (11.7–30.0)**	46.8 (20.5–73.1)	**43.9 (32.6–55.1)**
Diabetes Mellitus (%)	14.9 (5.4–24.4)	20.7 (1.8–39.6)	15.9 (8.2–23.6)
N° of Depressive symptoms	0.05 (-0.01–0.12)	**6.3 (5.9–6.7)**	0.07 (0.01–0.13)
Major Depression (%)	0 (0–0)	**99.4 (63.7–135.0)**	0.1(-0.1–0.4)
Hearing loss (%)	26.3 (17.8–34.8)	37.2 (8.8–65.7)	17.8 (10.3–25.3)
Alcohol units per week	**13.6 (8.6–18.6)**	9.3 (3.7–14.9)	**5.4 (3.1–7.6)**
OH excess (%)	15.5 (8.2–22.8)	15.3 (8.6–39.4)	5.6 (0.2–11.0)
Current smoker (%)	30.5 (22.6–38.5)	46.3 (13.4–79.2)	24.1 (15.6–32.5)
Physical activity per week (Mets)	**2932.8 (2281.0–3584.5)**	1559.7 (781.3–2338.2)	**1046.6 (750.5–1342.7)**
Physical inactivity (%)	**24.9 (15.8–34.0)**	49.0 (20.5–77.5)	**52.8 (40.1–65.4)**
**Men later life**	**High Metabolic Risk**	**High OH Intake**	**Physically Inactive**
Years of education	**3.3 (2.5–4.1)**	**9.1 (8.0- 10.2)**	**14.2 (13.32–15.1)**
Low education (< 7 years) (%)	**95.4 (64.- 126)**	**32.9 (20.8–45.0)**	**1.7 (-0.7–4.1)**
Systolic blood pressure (mmHg)	151.71(147.1–156.2)	142.4 (136.1–148.7)	145.6 (138.7–152.5)
High systolic blood pressure (%)	72.5 (41.9–103.1)	56.6 (38.6–74.7)	54.8 (29.9–79.8)
Body Mass Index (BMI)	29.3 (28.1–30.5)	**24.5 (23.8–25.1)**	29.7 (28.9–30.4)
Obesity (BMI > 30) (%)	44.6 (21.6–67.5)	**4.5 (1.4–7.7)**	45.6 (27.0–64.2)
Diabetes Mellitus (%)	31.0 (19.4–42.5)	24.2 (11.6–36.8)	27.3 (13.8–40.7)
N° of Depressive symptoms	0.4 (0.1–0.7)	0.3 (0.1–0.5)	0.35 (0.05–0.65)
Major Depression (%)	7.8(1.5–14.1)	2.9 (0.5–5.4)	4.3 (0.5–8.0)
Hearing los (%)	50.7 (27.0–74.4)	48.2 (31.2–65.3)	41.1 (17.5–64.8)
Alcohol units per week	2.6 (1.1–4.1)	**8.1 (5.4–10.8)**	2.4 (0.9–3.8)
OH excess (%)	1.0 (0.6–2.7)	6.1 (0.3–12.4)	**0 (0–0)**
Current smoker (%)	**4.8 (0.4–9.1)**	**25.5 (12.1–38.9)**	23.0 (0.5–45.5)
Physical activity per week (Mets)	**593.5 (389.7–797.3)**	**1922.3 (1244.4–2600.3)**	**285.1 (203.0–367.2)**
Physical inactivity (%)	63.7 (49.9–77.5)	50.5 (32.3–68.7)	78.8 (51.7–105.9)

The mean (95% confidence interval) is shown for continuous variables, for categorical variables the proportion (%) (95% confidence interval) is shown. *Clusters with statistically significant differences are bolded

### Clusters of dementia risk factors

**Fig. 1:**
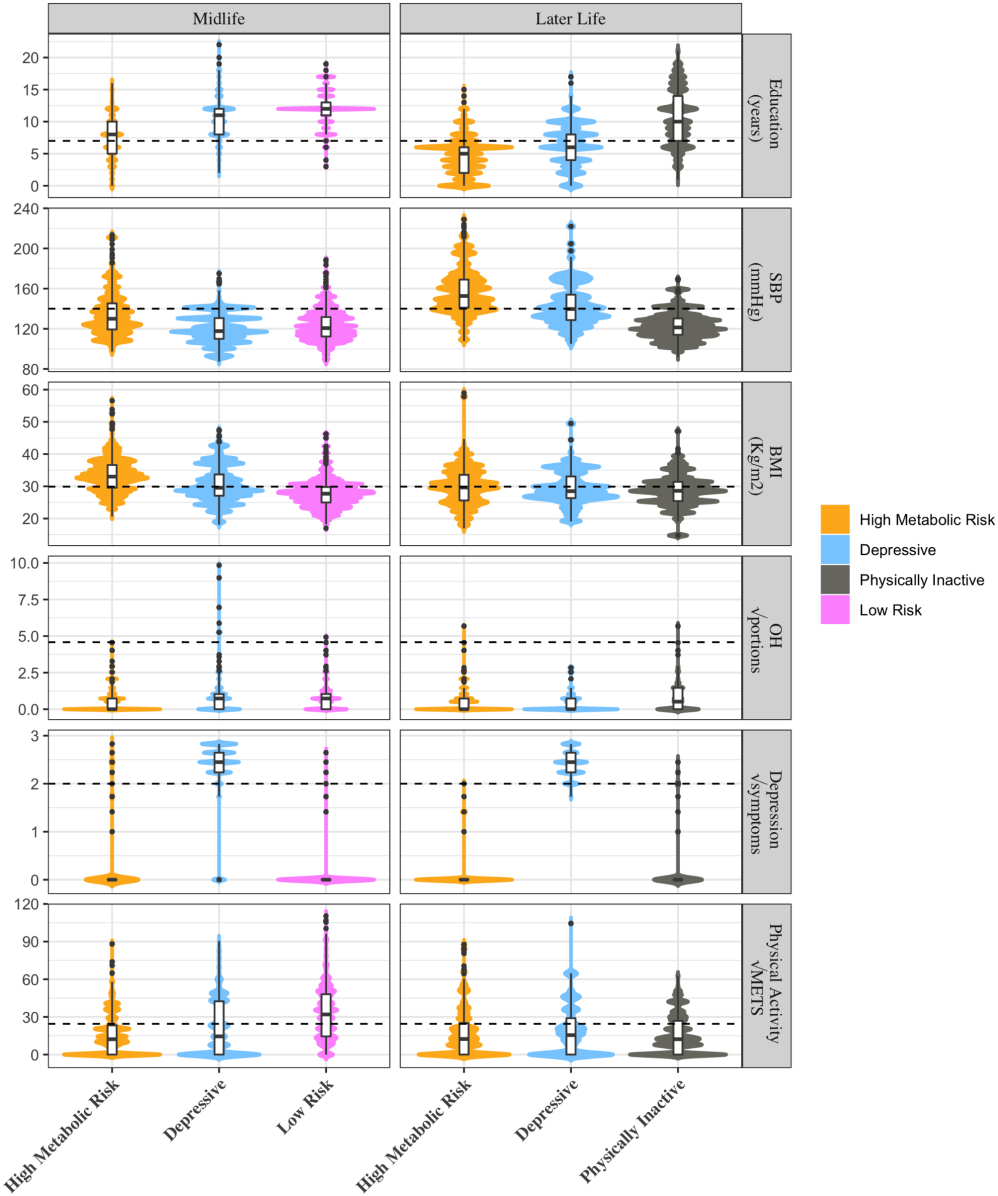
Clusters of dementia risk factors in women. Each of the 12 panels shows violin plots representing the scores of the 3 clusters (X-axis) within the 6 variables used for clustering with their respective measurement units in the Y axis (the horizontal line shows the cutoff point for considering that variable a risk factor for dementia). “√” indicates that the square root of the variable is presented

**Fig. 2:**
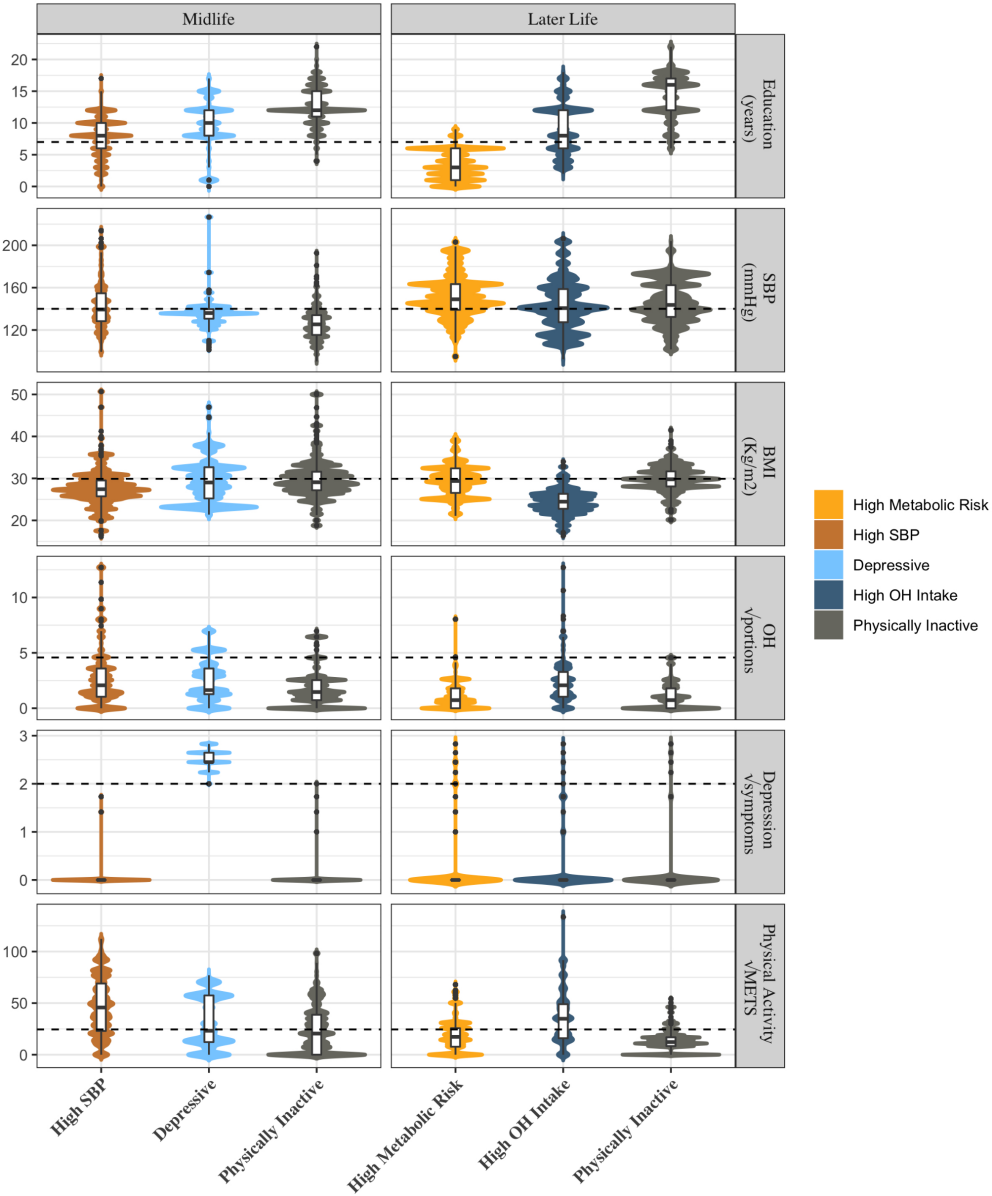
Clusters of dementia risk factors in men. Each of the 12 panels shows violin plots representing the scores of the 3 clusters (X-axis) within the 6 variables used for clustering with their respective measurement units in the Y axis (the horizontal line shows the cutoff point for considering that variable a risk factor for dementia). “√” indicates that the square root of the variable is presented

We identified three clusters with similar risk factors in each of the four sex/age groups, including (1) high metabolic risk or high systolic blood pressure (HSBP), (2) depressive symptoms, and (3) physically inactive clusters, while two additional clusters were found in specific sex/age groups (low risk and high alcohol intake clusters) (Figs. 1 and 2).

The “High systolic blood pressure” cluster was usually combined with high BMI, in which cases it was called the “High metabolic risk” cluster; it was the most frequent cluster, being the one with the largest proportion in women. In women in midlife, the other two clusters were a “Depressive” and a “Low risk” cluster. In women later-life, there was also a “high metabolic risk” and a “depressive” cluster, but instead of a “low risk,” there was a “physically inactive” cluster, representing 48%, 17%, and 35% of the population of women older than 65 years, respectively (Table 2). In men at midlife, there was a cluster with HSBP (without high BMI), a “Depressive”, and a “Physically inactive” cluster, representing 38%, 12%, and 50% of the population of men between 45 and 64 years old, respectively, while in later life, there was a “High Metabolic Risk”, a “Physically inactive”, and a “High alcohol intake” cluster, representing 36%, 27%, and 37% of the population of men older than 65 years, respectively (Table 3).

**Table 3:** Sensitivity analysis: comparison of additional risk factors

Women midlife	High metabolic risk	Depressive	Low risk
Monthly total family income (USD$)	**394.2 (347.1–440.7)**	533.9 (459.6–608.2)	588.6 (525.1–652.1)
Waist circumference (cm)	**101.3(99.7–102.9)**	93.9 (89.5–98.2)	90.27(88.7–91.8)
Obesity (Waist circumference) (%)	**90.0(76.1–103.8)**	68.2 (48.5–88.0)	**56.6 (43.8–69.5)**
Fasting glucose levels (mg/dl)	**109.2 (103.9–114.5)**	101.7 (95.5–107.9)	**94.08 (91.5–96.6)**
Metabolic syndrome (%)	67.8 (51.3–84.2)	52.9 (27.3–78.5)	40.3 (28.6–52.0)
**Women later life**	**High metabolic risk**	**Depressive**	**Physically inactive**
Monthly total family income (USD$)	348.9 (307.9–389.8)	370.1 (316.3–424.0)	**698.2 (439.3–957)**
Waist circumference (cm)	96.9 (94.5–99.3)	95.1 (91.8–98.4)	93.3 (90.5–96.0)
Obesity (Waist circumference) (%)	73.1 (60.4–85.8)	69.7 (49.2–90.2)	67.8 (51.7–83.9)
Fasting glucose levels (mg/dl)	103.3 (98.5–108.1)	126.0 (96.8–155.3)	103 (97.8–108.1)
Metabolic syndrome (%)	75.4 (56.3–94.5)	82.5 (50.7–114.3)	45.4(31.5–59.3)
**Men midlife**	**High systolic blood pressure**	**Depressive**	**Physically inactive**
Monthly total family income (USD$)	**505.3 (438.3–572.3)**	638.6 (469.5–807.6)	**1098.7 (767.9–1429.5)**
Waist circumference (cm)	96.1 (94.5–97.7)	99.6 (94.3–104.9)	99.7 (97.9–101.4)
Obesity (Waist circumference) (%)	23.7 (13.7–33.7)	43.6 (17.6–69.5)	30.7 (22.6–38.8)
Fasting glucose levels (mg/dl)	102.2 (98.4–106.0)	101.1 (94.4–107.7)	104.8 (99.8–109.8)
Metabolic syndrome (%)	58.2 (42.1–74.3)	82.8 (35.0–130.5)	67.1 (48.9–85.3)
**Men later life**	**High metabolic risk**	**High OH intake**	**Physically inactive**
Monthly total family income (USD$)	392.5 (322.3–462.5)	492.5 (360.1–624.7)	**759.5 (526.8–992.2)**
Waist circumference (cm)	101 (98.1- 103.8)	**88.6 (86.3–90.8)**	102.0 (95.9–108.0)
Obesity (Waist circumference) (%)	50.4 (26.4–74.4)	6.5 (2.6–10.4)	61.3 (36.6–86.0)
Fasting glucose levels (mg/dl)	105.9 (98.0–113.8)	101.5 (96.5–106.1)	105.0 (98.1–112)
Metabolic syndrome (%)	64.7 (33.1–96.3)	**28.5 (11.9–45.1)**	**80.5 (50.2–110.8)**

The mean (95% confidence interval) is shown for continuous variables, and the proportion (95% confidence interval) is shown for categorical variables. *Clusters with statistically significant differences are bolded

#### Cluster characterization and association with SDH

In general terms, the clusters in later life had a larger number of dementia risk factors than those in midlife, especially lower education and SBP, while obesity was more prevalent in midlife clusters.

##### High systolic blood pressure and high metabolic risk cluster

**Fig. 3:**
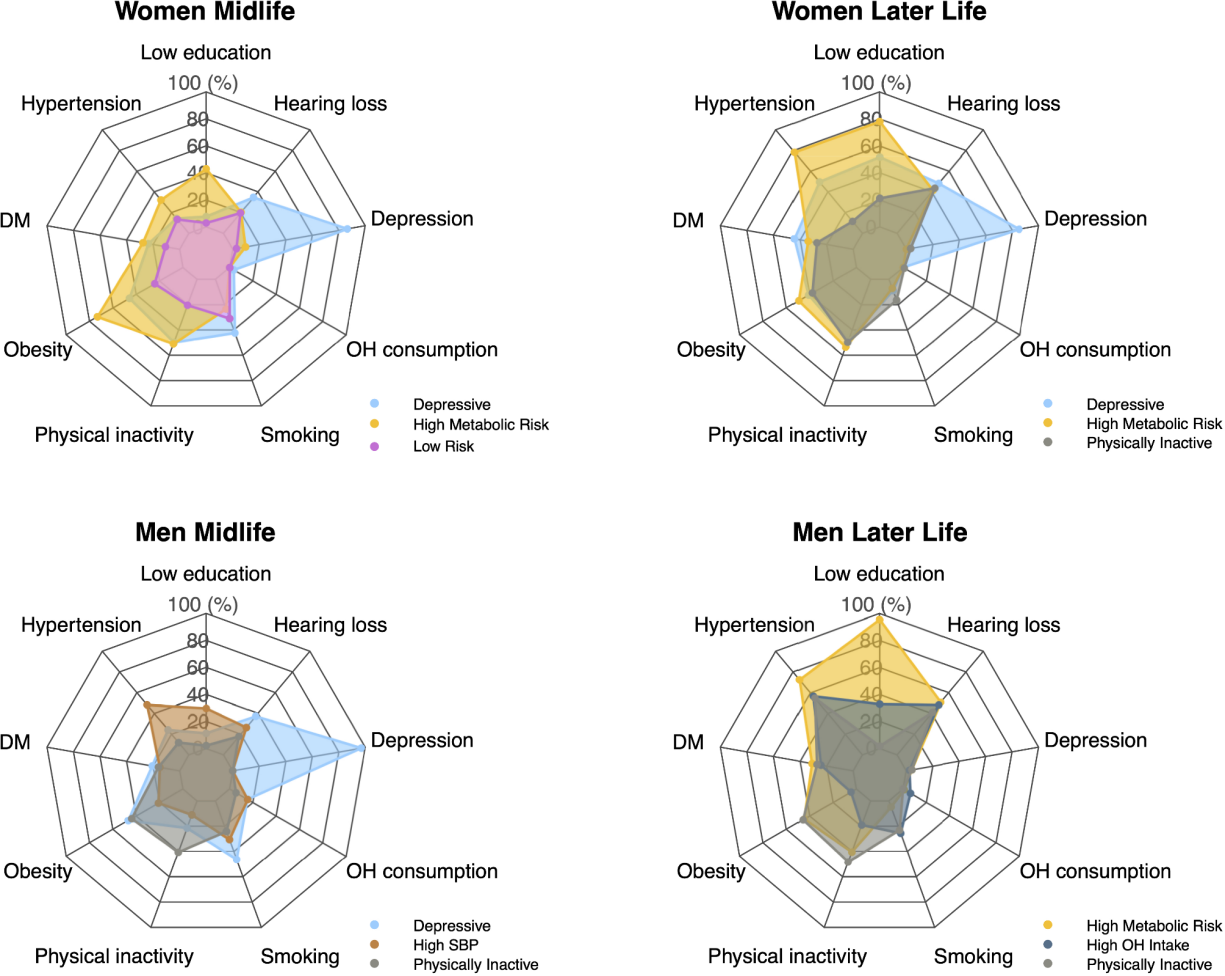
Prevalence of 9 dementia risk factors in each cluster. Each panel represents the sex/age groups. DM: diabetes mellitus, OH: Alcohol

In women in both age groups, HSBP was associated with HBMI, further being called the HSBP/HBMI or “High metabolic risk” cluster. This cluster had significantly higher levels of SBP than the other clusters in both age groups, with SBP being higher in later life than at midlife, while BMI was higher at midlife than in later life. This cluster had a significantly higher prevalence of obesity than the other clusters at midlife, as measured by BMI and waist circumference. Concerning behavioral risk factors, this cluster had very low physical activity levels, with more than 70% of physical inactivity in both age groups being significantly higher than in the low-risk cluster. In contrast, OH consumption was very low in both age groups (Table 2; Figs. 1 and 3, and e-Fig. 1 in suplementary material.).

**Fig. 4:**
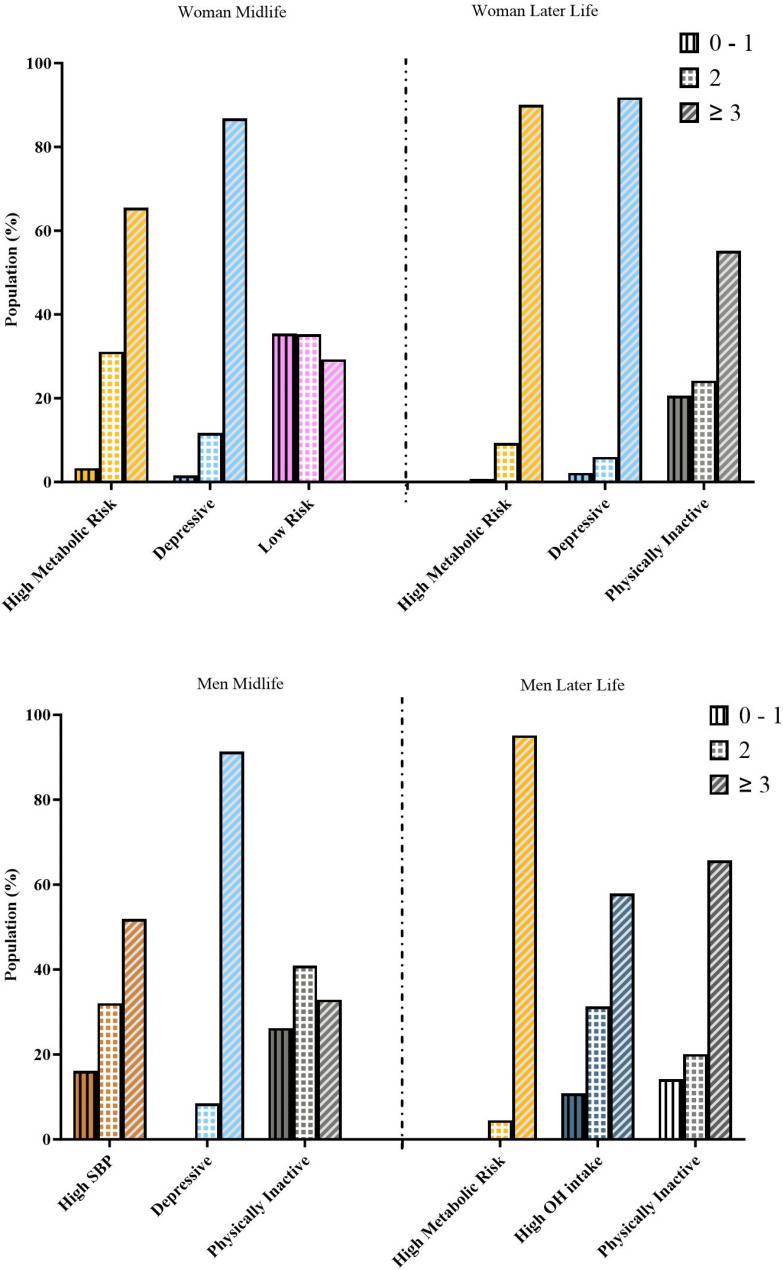
Number of dementia risk factors (0–9) in each cluster. Each plot represents sex/age groups. In X axis: are the Clusters, Y axis: the Population %. Each column’s pattern represents the number of dementia risk factors (0–1, 2 and ≥ 3)

As expected, the “high metabolic risk” cluster had a significantly higher prevalence of diabetes and higher levels of fasting plasma glucose than the low-risk cluster at midlife. Additionally, it had a high prevalence of metabolic syndrome. (Tables 2 and 3; Fig. 1). In addition, this cluster had a very high prevalence of multiple risk factors, with 65% having three or more dementia risk factors in midlife and 90% having three or more risk factors in later life (Table 3; Fig. 4).

In men, there were important differences between age groups; in midlife, the HSBP cluster had a low prevalence of obesity and the highest level of physical activity. In contrast, the HSBP/HBMI cluster in later life was associated with obesity and physical inactivity; thus, similar to women, it was called the high metabolic risk cluster (Table 2; Figs. 2 and 3). Cornering other dementia risk factors, it had higher OH consumption than the physically inactive group at midlife, low levels of depression, and average levels of hearing loss (Table 2; Fig. 4).

Furthermore, in men in later life, the high metabolic risk cluster had the highest prevalence of multiple dementia risk factors between clusters, with 95% of its population having 3 or more dementia risk factors (Table 3; Fig. 4).

The high metabolic risk cluster was consistently linked to the lowest educational attainment among all clusters (both age groups for women and men in later-life). Additionally, among women in midlife, the high metabolic risk cluster exhibited the lowest family income compared to other clusters. In midlife men, the hypertensive cluster had lower income than the cluster characterized by physical inactivity (Tables 2 and 3; Figs. 1, 2, 3 and 4).

##### Depressive cluster

Distinctively, this cluster had the highest prevalence of depressive symptoms among clusters, with the great majority of its population (> 90%) fulfilling the definition of a major depressive episode according to the DSM IV (Table 2). This cluster was present in women in both age groups and men of middle age.

In both sexes, it was associated with a high prevalence of metabolic risk factors and high rates of behavioral risk factors, with higher levels of obesity than the low-risk cluster and the highest proportion of OH excess in midlife women and higher blood pressure than the physically inactive women at later life. Likewise, its population had a high report of hearing loss (> 30% in both age groups) (Table 2; Fig. 3). Consequently, it had a high proportion of multiple risk factors, with 80–90% of the population having three or more dementia risk factors (e-Table 3; Fig. 4).

In the case of women, the depressive cluster exhibited distinct groupings in relation to SDH, which varied depending on the age group; it was associated with lower levels of education only among older individuals. However, for men, the groupings within the clusters did not show any discernible relationship with education or wealth.

##### Physically inactive cluster

This cluster was present in women later-life and in men in both age groups. In all of them, it was characterized by very low physical activity as the principal risk factor, with the lowest levels of physical activity between clusters in men in both age groups. It was also associated with HBMI but low HSBP, having a significantly higher BMI than the hypertensive cluster in men at midlife and the lowest prevalence of HSBP between clusters in midlife men and later-life women. It had a high prevalence of other metabolic risk factors, with a significantly higher prevalence of metabolic syndrome than the high OH intake cluster in men later-life (Table 3; Fig. 3). In addition, the physically inactive cluster had a relatively low prevalence of other dementia risk factors rather than HBMI (Fig. 4).

The physically inactive cluster had the highest educational level and the highest income between clusters in later-life and men in midlife. (Tables 2 and 3; Figs. 1, 2, 3 and 4).

##### High alcohol intake

This cluster was present only in men’s later-life. It had excessive alcohol consumption as the principal risk factor, together with a high smoking prevalence, having the highest alcohol consumption between clusters. It had the lowest BMI and waist circumference, associated with the highest levels of physical activity between clusters of men later-life. As expected, it had the lowest prevalence of metabolic syndrome. Additionally, it had a relatively low proportion of other dementia risk factors (Tables 2 and 3; Figs. 2–4 and e-figure 1 suplementary material). It was not clear whether the level of education or income played a relevant role in this group aggregation.

##### Low risk

This cluster was present only in women in midlife. It was characterized by the lowest obesity prevalence (both using BMI and waist circumferences) and the highest levels of physical activity between clusters. Accordingly, it had lower diabetes prevalence and lower fasting glucose levels than the high metabolic risk cluster. In addition, it had a very low prevalence of other dementia risk factors. (Figures 2, 3 and 4).

Concerning the association with SDH, it had a tendency for having a higher educational level (mean 12 years) and higher family income than the other clusters. (Tables 2 and 3; Figs. 1 and 4).

## Discussion

In a nationally representative sample of Chilean adults > 45 years old stratified into four groups by sex and age (midlife and later life), we show that six of the principal dementia risk factors (education, systolic blood pressure, body mass index, alcohol consumption, physical activity, and depressive symptoms) are grouped, generating three clusters of individuals who share similar risk factors in each sex/age group, with a total of five different clusters in the whole population > 45 years old. Remarkably, we showed that these clusters were distinctively associated with SDH. Taken together, the larger cluster corresponded to the “high cardiovascular risk clusters” that included the HSBP that was commonly associated with HBMI in women, defined as “high metabolic risk.” This cluster had characteristically fewer years of education and lower family income than the other clusters. The second largest group was the physically inactive cluster, which has relatively lower dementia risk, being only associated with HBMI; it had higher levels of education than the other clusters. The third largest cluster was the depressive cluster, which was more represented in women; high depressive symptoms, important multimorbidity, and variable association with SDH characterized it. Additionally, there was a cluster that was relatively healthy but with a risk of excessive alcohol consumption in men later-life and a low-risk cluster in women midlife that was associated with higher levels of education than the other clusters.

Our research findings suggest that in Chilean adults, lower levels of education are closely associated with an increased presence of cardiometabolic risk factors, particularly high systolic blood pressure, and a greater likelihood of experiencing multiple health conditions simultaneously. In contrast, higher educational levels are related to a lower prevalence of dementia risk factors and better control of SBP. Conversely, the clusters of behavioral risk factors were not related to educational levels, with physical inactivity being prevalent across populations of different educational levels.

The sensitivity analysis further validated the clusters, showing that the high metabolic risk cluster also had a higher prevalence of other metabolic risk factors and that educational levels were directly associated with total family income (Tables 2 and 3).

The association between lower educational level and high metabolic risk in our study is in accordance with a large amount of evidence that has shown an increased prevalence of cardiovascular risk factors, an increased risk of cardiovascular events, and cardiovascular death associated with a lower level of education [22]. An inverse association between years of education and cardiovascular risk factors has been shown [22, 23], principally with HSBP [15, 22, 23]. Furthermore, the association between lower education in childhood, later high cardiovascular risk factors in midlife, and future higher dementia risk in later life [24, 25] has been corroborated by the increased burden of cerebrovascular disease in low-educated adults, who have an increased risk of Aβ-negative subcortical vascular cognitive impairment [26].

The association between educational level and total family income was expected because, as a structural determinant of health, lower educational levels in childhood are associated with lower socioeconomic status in adulthood [15, 22, 23]. The strong association of lower education/lower income with adult HSBP, high metabolic risk, and high multimorbidity could be associated with multiple intermediary risk factors across the lifespan, such as unhealthy lifestyle (like physical inactivity in women) and higher exposure to unhealthy food and ambient toxins [14]. Another possible mechanism associating SDH with hypertension and worse health outcomes, in general, could be related to an allostatic overload due to the cumulative burden of chronic stress and life events of the more disadvantaged segments of society [14, 15]. Finally, lower education/ wealth is associated with a determined subculture, which could be related to lower healthcare awareness and/or difficulties in healthcare accessibility due to lack of time and/or low availability of healthcare providers where these persons live [22, 23]. Indeed, in the English Longitudinal Study of Aging, the increased dementia risk of the population with lower socioeconomic levels was mediated in 52% by the increased proportion of modifiable dementia risk factors for this subpopulation [27].

The depressive cluster distinctively presented a high number of depressive symptoms, with more than 90% of the cluster having more than 5 symptoms (Figs. 1 and 2; Table 2). It was also characterized by a very high prevalence of multiple dementia risk factors, having the highest proportion of multimorbidity in both sexes at midlife, with important rates of cardiovascular diseases in midlife and later life (Figs. 3 and 4). In fact, a synergistic bidirectional relationship between depression and cardiovascular diseases, principally diabetes [14], has been proposed, where each condition increases the possibilities of poor control and poor outcomes of the other. Additionally, individuals with depressive symptoms develop more medical symptoms ^28,^ increasing the possibility of overestimation bias in self-reports of health conditions. The association of depression with a high proportion of unhealthy habits found in Chilean adults has been described previously with physical inactivity, excessive alcohol intake, and smoking ^28,^ which could accentuate the risk of cardiovascular diseases.

There were different associations between the depressive cluster and the SDH in different sex/age groups, with lower educational levels in women later-life and men midlife but not in women midlife. This variability is in accordance with the fact that depression is associated with more diverse and complex factors, such as unfavorable experiences during childhood, traumatic events during the lifespan [29], and a strong genetic predisposition when it is present in midlife [30].

Concerning the dementia risk of the different patterns of multimorbidity, longitudinal studies using data from the UK [9, 13, 24] and Sweden [31] have shown that the hazard ratio is especially high in clusters of cardiometabolic [13, 31]and neuropsychiatric diseases [31]. HSBP has a higher hazard risk for dementia than behavioral cardiovascular risk factors [24]. Considering these longitudinal cohorts of multiple dementia risk factors [9, 13, 24, 25, 31, 32], the high metabolic risk cluster and the depressive cluster in the Chilean adult population had the highest dementia risk in the next 10–20 years because of the high hazard risk for dementia associated with HSBP and depression [11]. In addition, because of their high proportion of multiple dementia risk factors, 80 to 90% of their population had three or more dementia risk factors (Table 3; Fig. 4), which had been associated with a hazard ratio for dementia of 2.21 at all ages (95% CI 1.78–2.73) [9] but increased to an HR of 4.95 (2.54–9.67) when present at midlife [32].

Proposed targeted interventions for dementia prevention at a global population level should focus on increasing education levels as it is a pivotal social determinant of health [15–17]and on promoting a healthy lifestyle throughout the lifespan [12, 15, 24]. Considering the widespread obesity and diabetes epidemics across all socioeconomic levels, it is crucial to incentivize physical activity due to its inverse association with BMI presented in our study [14]. Lessons from successful anti-smoking campaigns have shown the effectiveness of strategies such as regulation, taxation, and incentives [17]. Similar approaches can be applied to promote active transportation, discourage car use, and implement taxation policies targeting ultra-processed food and sugary beverages [14].

In high-risk populations with a high proportion of multimorbidity, such as those in the high metabolic risk and depressive clusters, a biopsychosocial approach is recommended [14]. This approach involves multidisciplinary interventions encompassing mental health strategies such as psychotherapy and motivational interviewing, as well as biomedical pharmacological interventions with a special emphasis on controlling hypertension [14, 23]. Targeted campaigns should target the lower socioeconomic population, utilizing community-based and person-based approaches [16]. For example, periodic preventive health check-ups at workplaces, public places, or in conjunction with other civic compulsory procedures [14–16, 23]are recommended to increase the diagnosis of common conditions such as hypertension, obesity, and diabetes. Additionally, training community leaders as health promoters have proven effective in reaching those with lower socioeconomic attainment in certain low- and middle-income countries [9, 10].

### Strengths and limitations

As strengths, our study utilized diverse dementia-related variables, including metabolic, behavioral, mental health, and socioeconomic factors. These variables were measured using precise and reliable methods, with objective assessments for metabolic variables and validated questionnaires for behavioral, mental health, and social variables [18].

However, there are limitations to consider. The relative risk data for dementia risk factors primarily came from longitudinal cohort analyses conducted in North America and Europe, which may not fully reflect the ethnic and socioeconomic realities of our study population that may have different hazards for dementia associated with some risk factors, possibly related with the important associations between cardiovascular risk and SDH in our cohort [10]. Additionally, our data are cross-sectional, limiting our possibility of making causal inferences regarding social determinants of health. These limitations are common among studies conducted in Latin American and Caribbean regions [3–6]. Also, from the multidimensional scope of SDH, we used only the educational and socioeconomic dimensions, both considered structural SDH, but we did not include variables such as healthcare access, neighborhood, and social and community context [15]. Instead, we inferred these dimensions from the structural SDH used. Although the dementia risk factors included in our study align with those recommended by the Lancet Commission for Dementia Prevention, Intervention, and Care (2020) (11), some neurodegenerative diseases are underrepresented in this data. This may result in a bias toward risk factors that are more relevant to the specific etiologies Alzheimer’s disease, mixed dementia, and vascular cognitive impairment. Finally, our study slightly underrepresents men at midlife, but it does not seem to bias our results because they have similar cardiovascular risks as the included participants.

## Conclusions

Our findings highlight the significant role of educational level and family income in clustering dementia risk factors in the Chilean population; therefore, these associations should be evaluated in the rest of Latin America. Our results underscore the need for public health action plans to prioritize the effective management of the substantial burden of cardiometabolic risk factors, particularly among individuals with lower educational levels. Furthermore, our findings emphasize the importance of exploring the impact of mental health on other risk factors and the multidisciplinary approach that should be performed in these high-risk populations. Last, we emphasize the importance of tailored public health strategies that consider specific social determinants of health profiles, considering cultural and social barriers of the population rather than implementing generic campaigns.

## Electronic supplementary material

Below is the link to the electronic supplementary material.

Supplementary Material 1

Supplementary Material 2

## Data Availability

No datasets were generated or analysed during the current study.

## Abbreviations

Ch-NHS: Chilean national health survey
HSBP: High systolic blood pressure
BMI: Body mass index
HBM: High body mass index
HSBP/HBMI: High systolic blood pressure and high body mass index
SDH: Social determinants of health
DALY: Disability-adjusted life year
DSMIV: Diagnostic and statistical manual of mental disorders, 4th edition
OH: Alcohol
PAM: Partitions around medoids
