# Supplementary figures and images for: Modifiable dementia risk factors in Chilean adults are distinctively associated with social determinants of health. Cross-sectional study
Source: BMC Public Health. 2025 Mar 24;25:1117. doi: 10.1186/s12889-025-22220-6 (PMC11934555; doi:10.1186/s12889-025-22220-6)

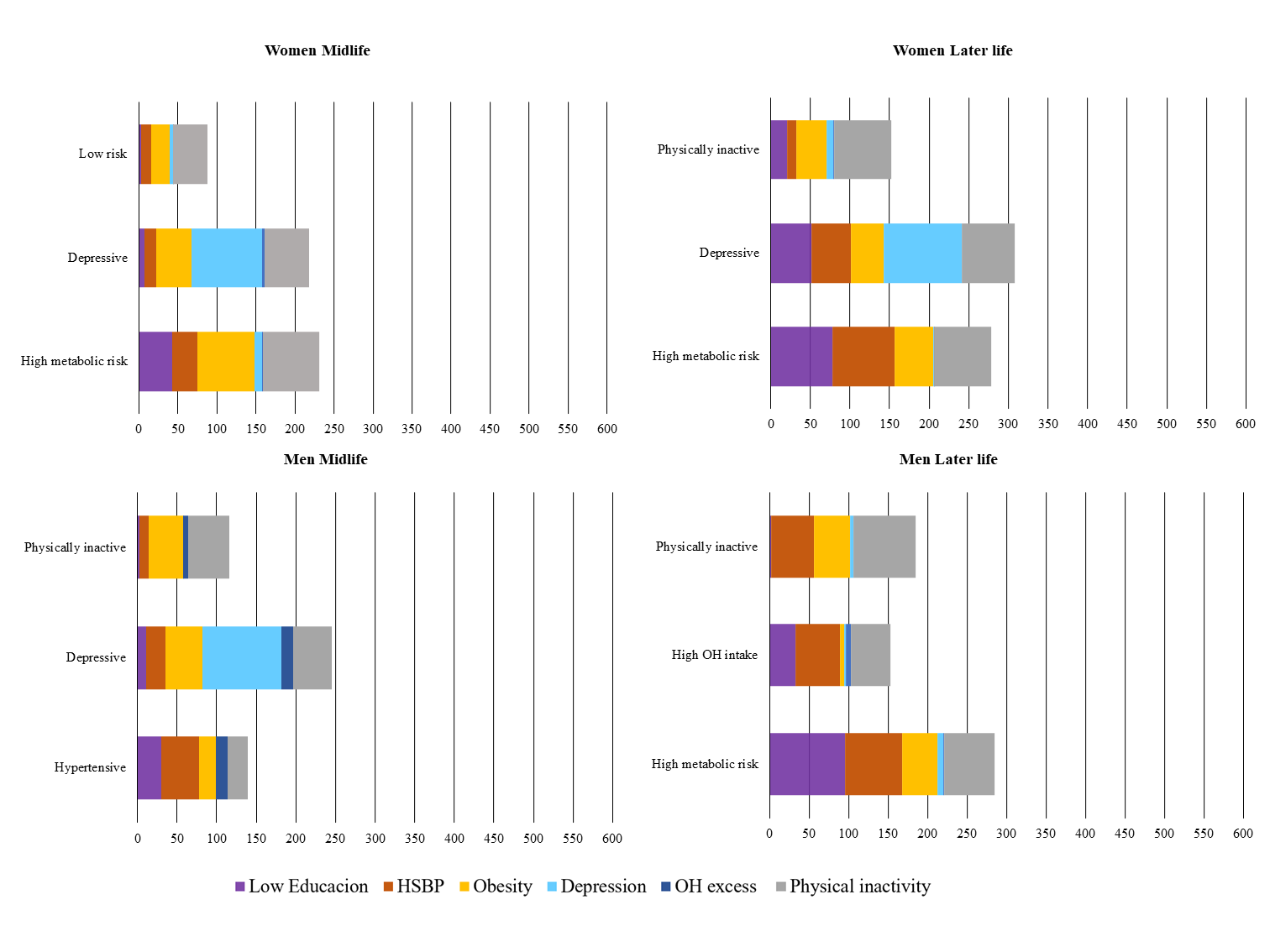

Supplement: Supplementary file 1 — Supplementary Material 1 [file 12889_2025_22220_MOESM1_ESM.png]
