# Supplementary material for: Modifiable dementia risk factors in Chilean adults are distinctively associated with social determinants of health. Cross-sectional study
Source: BMC Public Health. 2025 Mar 24;25:1117. doi: 10.1186/s12889-025-22220-6 (PMC11934555; doi:10.1186/s12889-025-22220-6)
Supplement: Supplementary file 2 — Supplementary Material 2 [file 12889_2025_22220_MOESM2_ESM.docx]

**E-Table 1. Definitions of eleven variables related with dementia risk**

| Variable | Measurements or Questions used in the Ch-NHS 2016-2017 | Continuous variable | Cut off for dementia risk factor |
| --- | --- | --- | --- |
| 1.- Years of education | 1.-What is the highest level achieved or current educational level?   1. What is the highest level achieved or current educational level? 2. Never attended. 3. Nursery school 4. Kindergarten (junior and senior kindergarten) 5. Prekinder / Kinder (Transition minor and Transition major) 6. Special Education (Differential) 7. Elementary or High School (Old System) 8. Basic Education 9. Humanities (Old System) 10. Scientific-Humanistic Secondary Education 11. Technical Commercial, Industrial or Teacher Training (Old System) 12. Professional Technical High School Education 13. Technical Higher Level (1-to-3-year courses) 14. Professional (careers of 4 or more years) 15. Postgraduate   2 -At that educational level, what was the last course you passed? | Years of education | Lower education: When not receiving more than primary education in early life (<7 years of scholarship) |
| 2.-Total family income (monthly USD) | What is the approximate liquid monthly income of the entire household, i.e., adding all household members' income?  1. Less than de $98,40 USD  2. From $98,43 to $169,10 USD  3. From $170,36 to $275,10 USD  4. From $275,13 to $373,50 USD  5. From $373,57 to $484,48 USD  6. From $484,48 to $606,86 USD  7. From $606,86 to $767,09 USD  8. From $767,17 to $965,27 USD  9. From $965,28 to $1299,83 USD  10. From $1299,83 to $1985,09 USD  11. More than $1984,74 | USD monthly income | Not used |
| 3.-Systolic blood pressure (mmHg) | The average of three measurements with the participant in a sitting position after 20 min. rest | Raw value of SBP in mmHg | **High systolic blood pressure (HSBP)** If systolic blood pressure (SBP) ≥140mmHg.  **Hypertension:** If there is HSBP and or diastolic blood pressure ≥90. |
| Hypertension  Hypertension  Therapy  (self report) | Have you ever been diagnosed with high blood pressure by a healthcare professional?  Are you undergoing any treatment recommended by a healthcare professional to control your blood pressure? | Not applicable | **Answer Yes**  **Answer Yes** |
| 4.1- Nutritional status 1 Body Mass Index (BMI) | Calculated by dividing weight (kilograms) by height (meters squared) that were objectively measured by trained personnel | Raw value of BMI | Obesity: BMI ≥30 |
| 4.2.- Nutritional status 2 Waist circumference | Measured in centimeters (cm) by trained personnel | Raw value in cm | Obesity >88 cm in women and  >102 cm in men |
| 5.-Glucose metabolismsm &  Diabetes Mellitus | Self-report and/or medical treatment for diabetes, and/or fasting blood glucose | Fasting glucose levels (Glycemia mg/dL) | **Answer Yes, and/or**  **Glycemia ≥126 mg/dL); (≥7.0mmol/L)** |
| 6.-Metabolic syndrome | Characterized by :1.-Elevated waist circumference (≥ to 80 cm for women and ≥ 90 cm for men), 2- Elevated blood pressure (≥ 130/85 mmHg), 3.-High fasting plasma glucose levels (≥100 mg/dl), 4- Diabetes under treatment, 5- Hypertensive patients on treatment.7- Triglycerides≥150, 8.- HDL Cholesterol<50. | Not applicable | **Having 3 or more characteristics** |
| 7.-Depressive symptoms | Assessed with the Composite International Diagnostic Interview short form for depression (CIDI-SF) by a trained interviewer.   1. In the past 12 months, have you had two consecutive weeks or more where you felt sad, down, or depressed? 2. During those two weeks or more, did you lose interest in most things (such as hobbies, work, or activities) that are usually enjoyable to you? 3. During those same two weeks, did you feel more tired or have less energy than usual? 4. Did you lose or gain weight unintentionally, or did you stay about the same weight? 5. During those two weeks, did you have more trouble than usual falling asleep? 6. During those two weeks, did you have more difficulty than usual in concentrating? 7. People sometimes feel bad about themselves, that they are worthless, that they are not good enough. During those two weeks, did you feel that way? 8. During those two weeks, did you think a lot about death, whether it was your own, someone else's, or death in general? | For severity the presence of depressive symptoms was count from 0 to 8. | **Suspected Major depression** in the last year according to the fourth edition of the Diagnostics and Statistical Manual of Mental Disorders (DSM IV) |
| 8.-Hearing loss | Do you think you hear normally by both ears? Are you able to follow a TV program at a volume acceptable to others? Are you able to follow a conversation of three or more people? | Not applicable | Hearing loss: Self-reported by the negative response to any of the questions |
| 9.-OH consumption | Measured using two questions of the validated Chilean version^22^of the Audit questionnaire: When you drink alcohol, how many drinks (units) do you usually have on average per day? How often do you drink any alcoholic beverage? The results were shifted-up 20.3% to match results from the apparent per-capita | OH Units per week | Alcohol excess: (>21 units/week) alcohol consumption. |
| 10.-Current smoker | Self-reported by the affirmative response to: Do you currently smoke cigarettes? | Not applicable | Yes |
| 11.-Physical activity | Assessed with the Global Physical Activity Questionnaire version 2 (GPAQ), previously validated in LAC countries. We used the total physical activity in the last week. | METS (Kcal/Kg/min) | **Physical inactivity:** Defined when scored < 600mets per week. That is classified as low physical activity according to the GPAQ |

|  | **Women Midlife** | | **Women Later Life** | | **Men Midlife** | | **Men Later Life** | |
| --- | --- | --- | --- | --- | --- | --- | --- | --- |
|  | **Included** | **Excluded** | **Included** | **Excluded** | **Included** | **Excluded** | **Included** | **Excluded** |
| **N** | 2001107 | 249968 | 837765 | 153659 | 1726082 | 421855 | 654252 | 92814 |
| **%** | 88,9 | 11,1 | 84,5 | 15,5 | 80,36 | 19,64 | 87,58 | 12,42 |
| **Low education** | 19.24 (19.2 - 19.3) | **23.44 (23.3 - 23.6)** | **53.44 (53.3 - 53.5)** | 45.96 (45.7 - 46.2) | **13.87 (13.8 - 13.9)** | 9.32 (9.2 - 9.4) | 46.92 (46.8 - 47) | 47.13 (46.8 - 47.5) |
| **Hypertension** | 24.67 (20.7 - 28.6) | 41.3 (24.7 - 57.8) | 31.24 (24.5 - 37.9) | 28.14 (13.7 - 42.6) | 29.73 (22.1 - 37.3) | 27.27 (16.1 - 38.4) | 18.82 (13.9 - 23.8) | 8.04 (0.9 - 15.1) |
| **Hypertension**  **therapy** | 15.73 (12.8 - 18.5) | 27.52 (15.6 - 39.4) | 17.48 (12.6 - 22.4) | 18.84 (5.6 - 32.1) | 21.28 (14.5 - 28.1) | 19.03 (10.8 - 27.3) | 14.96 (10.6 - 19.3) | 6.78 (-0.3 - 13.8) |
| **Audition loss** | 24.05 (24 - 24.1) | 24.31 (24.1 - 24.5) | **43.34 (43.2 - 43.4)** | 30.57 (30.3 - 30.8) | **23.43 (23.4 - 23.5)** | 19.49 (19.4 - 19.6) | **47.24 (47.1 - 47.4)** | 35.67 (35.4 - 36) |
| **Smoking** | **31.33 (31.3 - 31.4)** | 17.4 (17.3 - 17.6) | **10.72 (10.7 - 10.8)** | 4.79 (4.7 - 4.9) | 29.21 (29.1 - 29.3) | **39.3 (39.1 - 39.4)** | **17.43 (17.3 - 17.5)** | 12.8 (12.6 - 13) |
| **Depression** | **28.52 (28.5 - 28.6)** | 19.02 (18.9 - 19.2) | **15.54 (15.5 - 15.6)** | 7.23 (7.1 - 7.4) | 11.48 (11.4 - 11.5) | **12.68 (12.6 - 12.8)** | 3.57 (3.5 - 3.6) | **6.98 (6.8 - 7.2)** |
| **Physical Inactivity** | **39.07 (39 - 39.1)** | 36.49 (36.3 - 36.7) | 50.41 (50.3 - 50.5) | **52.87 (52.6 - 53.1)** | 26.25 (26.2 - 26.3) | **27.36 (27.2 - 27.5)** | 33.88 (33.8 - 34) | **75.33 (75 - 75.6)** |
| **Diabetes** | 8.89 (6.7 - 11.0) | 15.26 (5.2 - 25.2) | 12.73 (7.9 - 17.5) | 7.92 (3.0 - 12.8) | 11.14 (7.4 - 14.9) | 11.32 (5.3 - 17.3) | 11.94 (5.3 - 18.6) | 5.45 (-1.1 - 11.9) |

**E-Table 2. Comparison between included and excluded participants**

Proportion (±95% confidence interval) is shown. * **statistically significant differences are bolded.**

**E-Table 3· Clusters of dementia risk factors, weighted numbers and proportions.**

| Sex/ Age group | Clusters of dementia risk factors | Chilean NHS  weighted Numbers (%) |
| --- | --- | --- |
| Women Midlife | High metabolic risk | 747,641 (37) |
|  | Depressive | 553,412 (28) |
|  | Low risk | 700,054 (35) |
|  | total | 2,001,107 (100) |
| Women later life | High metabolic risk | 398,657 (48) |
|  | Depressive | 141,409 (17) |
|  | Physically inactive | 297,699 (35) |
|  | total | 837,765 (100) |
| Men Midlife | High systolic blood pressure | 664,283 (38) |
|  | Depressive | 204,189 (12) |
|  | Physically inactive | 857,610 (50) |
|  | total | 1,726,082 (100) |
| Men later life | High metabolic risk | 234,764 (36) |
|  | High OH intake | 241,918 (37) |
|  | Physically inactive | 177,570 (27) |
|  | total | 654,252 (100) |
